# Barriers and enablers in the use of respite interventions by caregivers of people with dementia: an integrative review

**DOI:** 10.1186/s13690-018-0316-y

**Published:** 2018-11-22

**Authors:** Marie-Conception Leocadie, Marie-Hélène Roy, Monique Rothan-Tondeur

**Affiliations:** 1 School of Health Sciences, HES-SO University of Applied Sciences and Arts Western Switzerland, Avenue de Champel 47, CH 1206 Geneva, Switzerland; 2 Mont Champagnat Residence, CIO 7141 Royal Avenue Château-Richer, Quebec, GOA 1N0 Canada; 3 University of Paris, 13 Sorbonne Paris Cite, Nursing Sciences Research chair, Laboratory Educations and Health Practices (LEPS), (EA 3412), UFR SMBH, F-93017 Bobigny, France; 4 Assistance Publique – Hôpitaux de Paris, Nursing Sciences Research Chair Paris, Bobigny, France

**Keywords:** Caregivers, Alzheimer’s, Dementia, Respite, Literature review

## Abstract

**Background:**

Due to the increase in the number of people with dementia, relatives often provide in-home care. This care constitutes a cornerstone of the healthcare system, and maintaining these caregivers’ well-being is therefore of paramount importance. Although respite interventions are generally considered an effective support system, they tend to be underutilized. The aim of this integrative literature review is to highlight the factors that promote and impede the use of respite interventions.

**Methods:**

Searches were conducted on the PubMed and CINAHL databases for studies of respite interventions from 1980 to 2016, and they yielded 51 articles of relevance.

**Results:**

Analysis of these articles revealed modifiable and immutable factors that influence the use of respite. The most cited topic categories in the literature were attributes of respite services and workload managed by caregivers, which is characterized by the onset of burden.

**Conclusion:**

The factors promoting or impeding the use of respite interventions identified by our analysis highlight the need to adapt respite service attributes and use caregivers’ skills to foster the partnership between healthcare teams and caregivers and to ensure the accompanying dyad’s quality and safety.

**Electronic supplementary material:**

The online version of this article (10.1186/s13690-018-0316-y) contains supplementary material, which is available to authorized users.

## Background

Close to 35.6 million individuals globally are affected by dementia, and this number is set to double by 2030 and to triple by 2050 (reaching 115.4 million) [1, 2]. The high rate of cognitive degenerative diseases that accompanies the increase in the aging population [3] therefore represents a major international public health issue [2].

Due to limited progress in the development of therapies, dementia is still an incurable chronic disease. However interventions exist to support people with dementia and their families to reduce disruption of their lives and maintain their quality of life. Support from relatives is a cornerstone of the healthcare and the economic systems [4, 5]. In this review, the term “caregiver” refers to the primary caregiver; it does not include the health professionals who take care of people with dementia. The caregiver is defined as a primary caregiver “who is directly involved with an individual in need of care and who provides for that individual’s daily needs in an informal, non-professional manner. A caregiver is someone who assists an individual who is disabled or having difficulty in completing daily activities and who ensures their safety and connection with society. Family members, friends, and neighbors can all be caregivers” [6]. Several studies have shown that caring for people with dementia can have positive implications for caregivers. Therefore, by providing care, they may feel a sense of mastery and accomplishment that leads to personal growth and a sense of reward and satisfaction [7]. These positive feelings vary depending on various factors such as education, the duration of the caregiving, and the level of social support [8]. Despite these positive feelings, taking care of people with dementia can increase distress levels among caregivers who report poor health and a lack of family support [9]. On the other hand, the feeling of being prepared and a level of trust reduces the level of distress [10]. Caregivers are involved in this task on a daily basis, and they consequently feel overburdened at times [11–16]. The burden that caregivers feel can be defined as the combined set of social, emotional, psychological, physical, and financial consequences [17]. The overload due to the daily demands placed on caregivers can lead to their mental exhaustion.

The needs of caregivers can be met by having adequate information, training, recognition, proper organization regarding care, and above all respite from the demands placed on them [9]. Indeed, respite is essential for caregivers of people with dementia although the available respite programs appear to be underutilized [18, 19]. It is therefore important to identify the factors that promote or impede the use of respite services.

The support programs or caregivers have been progressively deployed to meet their needs. Interventions that provide information and training were the first to be introduced, followed by discussion groups providing psychological support. A genuine need has clearly emerged to develop specific interventions to provide respite for caregivers [20].

“Respite in the broad sense of the term is defined as an interruption in an absorbing or constraining occupation; rest”; “a momentary stop, the suspension of something painful or suffering.” [21]. Several types of respite interventions have been devised. Each separately addresses caregivers’ needs. Respite benefits can be provided during the day or night, at home or away from home, for a short period or on a daily basis in a daycare facility [22, 23]. They can be scheduled ahead of time or on short notice [24]. Respite decreases feelings of loneliness, anxiety, depression, and the sense of “burden” that caregivers often feel, thereby leading to an improvement in their behavior and their quality of sleep [25–27]. Respite also improves the relationships, family bonds, and behaviors of the person with dementia [28]. Certain programs have long-term beneficial effects on caregivers’ health [29].

Several types of studies have been conducted to assess the factors that influence the use of respite services for people with dementia. These studies are qualitative systematic reviews focusing exclusively on qualitative studies [30], a narrative synthesis of the literature that focuses only on non-use factors of respite services [31], and a review of the literature on the use of respite services but without the inclusion of mixed methods studies [32]. The purpose of this integrative review is to provide a different and specific method for summarizing empirical or theoretical literature to provide a more complete understanding of a healthcare problem or phenomenon [33]. This study was to target the non-use factors and the use factors of respite services by investigating studies with various methodologies.

## Methods

### Search strategy

The aim of this integrative review was to identify the barriers to and the factors promoting the use of respite interventions by caregivers for people with dementia. The results should help devise guidelines that policy and/or executive bodies can use for the implementation of support interventions.

An integrative review was conducted based on Whittemore and Knafl’s (2005) recommendations, and it involved five stages: 1) identification of the issue, 2) a literature search, 3) evaluation of the data, 4) analysis of the data, and 5) presentation of the results.

The integrative literature review is defined as a comprehensive systematic search method that includes all scientific studies. The theoretical and empirical elements are taken into account and analyzed in order to provide a complete understanding ensuring a systemic vision of the subject treated.

The search for relevant documents was carried out using PubMed and CINAHL databases. The MESH terms were “caregivers,” “Alzheimer,” “dementia,” and “respite.” The various terms were combined using Boolean operators to obtain a search equation (Additional file 1).

Two researchers carried out the article selection and analysis stages to ensure cross-checking and controls. Using the results in the various databases, we performed the first selection by restricting the languages; then we perused the titles and abstracts while using the criteria for inclusion and noninclusion. Elimination of duplicates was performed at the same time. Reading of the full articles then allowed for evaluation of the scientific studies’ methodological quality and the final selection.

### Study inclusion and exclusion criteria

The included articles were selected based on the following criteria: caregivers for people with dementia, respite services (caregiver respite in an institution or at home), and factors promoting or impeding the use of respite. The articles were published from 1980 to 2016, and they were written in French, English, Portuguese, and Spanish. Primary studies of a qualitative, quantitative, or mixed nature derived from the scientific literature were selected. To provide experts’ knowledge, the gray literature was also consulted. Studies conducted in hospital care but not matching care for people with dementia were excluded.

### Study quality assessment

Two researchers independently evaluated the methodological quality of the preselected studies with the “mixed methods appraisal tool” (MMAT) checklist [34]. This operational grid is composed of several items (Additional file 2). The quality grid allowed for a score ranging from 0 to 4, with the highest scores indicating better quality. Scientific studies with a score of 3 or 4 were selected. The scientific studies that did not meet the methodological criteria were not retained. When the evaluations were discordant, the two researchers discussed the discrepancy to reach a consensus. The consensus factors were based on the 19 items in the MMAT evaluation grid and on the levels of evidence Tavares et al. (2010) proposed for gray literature documents [34, 35]. In the end, 51 articles were selected with a moderate level of concordance (Kappa = 0.55).

### Data extraction strategy

After the article selection, a descriptive and analytical reading was carried out, and a summary table of the selected articles was generated. The analysis of the studies was carried out according to the principles recommended by Miles, Huberman, and Saldaña (2014). An encoding using the MAXQDA 12 software analysis tool allowed for a regrouping of subthemes in a pattern group and determination of the theme categories [36]. The data was coded in two stages. During the first coding cycle, codes that represented symbolic descriptive or deductive information were assigned. These codes were allocated to large parts of the participants’ statements, and they promoted the grouping of data segments. Then a second coding cycle promoted the identification of “pattern codes” to group these summaries into a smaller number of thematic categories. In the end, a data matrix was produced (see Fig. 2), allowing an analysis of the themes and a summary of the salient points guiding the discussion. The discussion was written in light of the current political context and the state of current scientific knowledge.

## Results

### Study selection

**Fig. 1:**
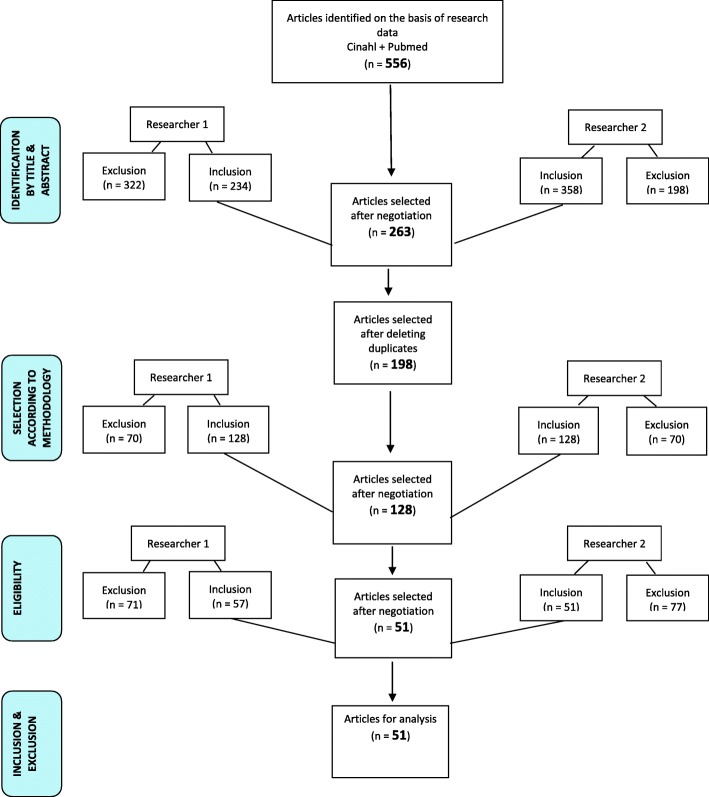
Flowchart of the selection procedure for the integrative literature review

A total of 556 articles were identified based on the selected keywords. After sorting, 51 articles were retained for the integrative literature review (Additional file 3). Of these studies, 22 were quantitative, 13 were qualitative, and one was mixed. To add expert knowledge on the subject, we added 13 expert opinions and 2 theses (see Fig. 1).

### Synthesis

**Fig. 2:**
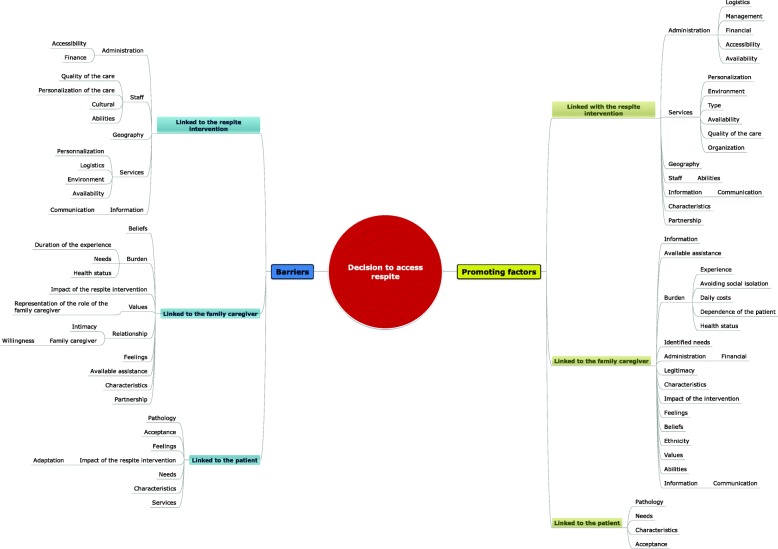
Thematic matrix

The results regarding the factors promoting and impeding the use of respite interventions were identified and regrouped into three categories: in connection with the respite interventions, with caregivers, and/or with the person with dementia. Several themes and subthemes emerged from these three categories (see Fig. 2).

To guide activities for caregivers, the theme categories were classified based on two broad factors, namely changeable factors and immutable factors. The changeable factors are defined as elements that can be subject to an action. By contrast, the immutable factors are unchangeable elements (see Table 1). With the software MAXQDA 12, the occurrence of themes and studies’ quotes were calculated from the codification.

**Table 1:** The occurrence of changeable and immutable factors promoting or impeding the use of respite

Factors	Categories	Number of studies	Occurrences of categories
Changeable factors	Beliefs ♣	13 [37, 41, 42, 52–56, 61, 66, 69, 70, 76]	25
	Feelings ♣♦	9 [41–43, 50, 53, 55–58]	19
	Burden (load ♣, pathology ♣♦, need of people with dementia♦)	31 [37–39, 42–44, 47–49, 51–59, 61–70, 75, 77, 96]	93
	Available assistance ♣	6 [37, 42, 47, 51, 54, 56]	18
	Acceptance ♦	7 [42, 49, 52, 56, 61, 69, 86]	12
	Communication ♣♠	13 [40, 42–44, 49, 50, 53, 54, 56, 70–73]	37
	Attributes of the respite interventions (administration, geography, service, staff)♠	40 [37, 39–45, 48, 49, 51, 53–59, 61, 65, 66, 68–73, 75–87]	239
	Legitimacy ♣	6 [48, 49, 51, 60, 61, 68]	6
	Partnership ♣♠	9 [39, 51, 57, 58, 68, 71, 76, 83, 85]	17
	Impact of the respite ♣♦	4 [42, 51, 56, 86]	4
	Abilities ♣♠	12 [49, 50, 54–56, 59–61, 64, 65, 87, 88]	38
Immutable factors	Ethnicity ♣	4 [37–40]	6
	Characteristics ♣♦♠	15 [38, 39, 41–45, 47–49, 56, 66, 67, 77, 83]	29
	Values ♣	9 [42, 48, 50, 51, 56, 59, 60, 65, 87]	24
	Relationship ♣	9 [39, 51, 57, 58, 68, 71, 76, 83, 85]	17

♣ Factor related to caregivers; ♦ Factor related to people with dementia; ♠ Respite service factor

In Table 1, the immutable factors are represented by elements on which the actions of health professional, decision makers, and politicians have little influence. Characteristics such as age, gender, ethnicity, values based on education, and history of the dyad are elements to which the health domain must adapt. Conversely, health actions can influence changeable factors. For example, the belief that respite services are not appropriate for people with dementia may depend on actions being taken by the health field for caregivers that can result in change.

For a question of presentation and meaning, some themes have been gathered below.

#### The factors referred to as “immutable”

##### Ethnic factors

Ethnic factors have a substantial impact on the use of respite [37–40], as various ethnic groups’ cultural attitudes can influence the decision to seek respite. For example, the feeling of guilt in relation to respite was higher for individuals of Caucasian descent and African-Americans than for Hispanics/Latinos [41].

##### The characteristics of caregivers and of their relatives facing the disease

Age, gender, and kinship can influence respite. Being the wife, an elderly caregiver, or a child of the relative facing the disease promotes the use of respite [38, 39, 41–46]. Being a spouse or male impedes the use of respite [38, 39, 43, 47–49]. Male caregivers believe that external respite interventions at home are not suitable for men. This belief can be indicated by stereotypical beliefs that men’s activities are different from those of women [49]. Beliefs, values, relationships, and feelings of the caregiver and the person with dementia also influence the use of respite and therefore should be taken into account.

##### Values (importance or interest carried to somebody, something, phenomenon or event) and the relationship of the dyad

Caregivers’ values may prevent them from accessing respite interventions because they fail to recognize that they have the right to a period of rest [48]. When family members, including the person receiving the care, disapprove of the respite [42], the caregiver will be unlikely to take a break from their care duties. To avoid conflict, caregivers often prioritize the wishes of their relative facing the disease [50]. In some cultures, the role of caregiver is seen as one of sacrifice and duty, which is a barrier to the use of respite [51]. The dyad’s existing relationship can sometimes be disrupted by the arrival of a new caregiver who calls into question the access to respite, which can result in its termination [37]. In that case, the medical or paramedical staff must reconsider the care based on new needs.

#### The factors referred to as “changeable”

##### Beliefs (certain opinions which, without being religious, have the character of an intimate conviction)

Caregivers believe that the use of formal respite interventions leads to financial concerns [37] and loss of control over the care provided to the relative with the disease [52]. These beliefs are barriers to the use of respite services. Furthermore, they deem that respite care is intended for the disease’s final stage not the early part of dementia care [53] because the patient may still be able to cope with his or her situation [42] and because it stigmatizes the people with dementia [54]. The caregivers believe that respite is disruptive to the patient due to the change in their environment [55]. Doubts regarding the quality of the care and the belief that respite may promote a decline in the health of the relative with the disease [52, 55] may increase the level of distrust in nighttime respite. Significant concern exists that the patient may be subject to abusive behavior from his or her carer [46]. Accepting an outsider into a caregiver’s home is therefore sometimes a barrier to the use of respite [37, 42, 56]. Respite services are most often used when caregivers believe that the respite promotes better care and that it delays hospitalization, especially for people with dementia and when their health is in jeopardy [55, 56]. Some beliefs cause emotions and feelings such as fear or guilt, which may also influence the use of respite.

##### Emotions/feelings

When the well-being of people with dementia is entrusted to another person, the sadness and the abandonment the patient feels can lead to feelings of betrayal, guilt, insecurity, and fear among caregivers that can change the level of use of respite services [42, 43, 46, 50, 53, 55, 57, 58]. However, when the person with dementia does not feel abandoned and the caregiver is aware that respite is necessary for his or her own health, the decision to seek respite care is simpler [48, 59, 60]. This decision will nevertheless be weighted according to the caregiver’s workload.

##### The burden (the load, pathology, and the needs of the relative with the disease), available assistance, and acceptance

A caregiver who has provided support for less than 3 years will be less inclined to use respite interventions than a caregiver who has provided care for more than 5 years [42, 61]. The use of respite depends on whether the caregiver has already felt overloaded [37, 54]. Indeed, caregivers who have busy daily schedules with little time to take care of their relatives on their own due to other obligations are more likely to use respite interventions [42, 43, 47, 48, 59, 62–64]. Moreover, caregivers that suffer from depression, exhaustion [48], anxiety [37], stress, or a disease [46, 51, 54, 59], are more willing to seek respite.

Nevertheless, if the respite time is used for non-recreational daily tasks and the feeling of burden is minimal, caregivers tend not use respite services [52, 65]. In addition, the number of co-morbidities of people with dementia and the presence of a behavioral disorder when returning from the establishment does not favor their use [58, 66].

Behavioral issues, an advanced stage of the disease, and cognitive impairments can promote or restrict the use of respite. A health status of people with dementia that enhances the need for supervision and socialization, such as nutritional issues and a major disability, are considered favorable factors for the use of respite services [37, 39, 46, 47, 49, 54, 55, 66, 67]. However, lengthy physical preparations with a need for extensive services are barriers to the use of respite [44, 68]. If taking care of people with dementia is considered a heavy burden, caregivers will prefer to use an informal respite solution and backup from family or friends [42, 56] to allow the patient to stay at home in a reassuring environment. The use of formal and informal respite varies with the acceptance of help by the caregiver and the person with dementia [42, 46, 49, 69]. However, to access respite services, families need to be informed.

##### Communication and access to information

Communication can represent a promoting factor and a barrier to respite. Lack of knowledge of the network linked with respite (e.g. services, conditions for admission, and caregivers’ degree of involvement) and the difficulties in accessing accurate information have been identified as barriers to the use of respite [42, 44, 46, 50, 53, 70–72]. The existence of explanatory training regarding respite (e.g. the teams’ expectations and limitations) and of a centralized source of information facilitate access to respite services [43, 49, 71, 73]. Moreover, a marketing strategy (e.g. multisite communication, brochures, journal publications, radio, local television outlets, visits to the facilities, newsletters, and advertising in the language common to the various areas) were identified as positive elements [53, 70], as they allow the information to reach a wide audience. Despite access to information and good communication, the effectiveness of respite time will be influenced by the attributes of respite care [74].

##### The attributes of the respite interventions (administration, geography, services, and staff)

Geographical access, administrative factors, staff, and the benefits provided by the services were important attributes influencing the use of respite. Living in a large urban area, being geographically removed from a respite service, or having a limited number of expert nursing care facilities were barriers [42, 66, 70]. By contrast, living in a rural area was a favorable factor [39]. At the administrative level, access to interventions was considered too complex and non-systematic, and it was also limited by the financial aspect (income, social security) [42, 70]. The centralization of administrative procedures makes it easier to access complicated respite interventions [37, 44, 53, 57, 70–72, 75]. Furthermore, being covered by the social security system and having adequate finances promote the use of respite [40, 66].

Better management as a result of working as part of a network and the availability of health professionals to clarify what may be expected from the respite interventions are paramount [75]. Caregivers expect personalized arrangements that are tailored to their needs and those of their family [39, 53, 76, 77]. Arrangements that are not personalized do not promote the use of respite [46, 49, 53, 68, 78]. Inflexibility in interventions and, for example, the unavailability of nighttime care are barriers [42, 53, 70, 71, 76, 78]. On the other hand, flexibility regarding the dyad’s needs, schedules, and programs appears to be a promoting factor [42, 70, 79, 80]. Personalization of care must be derived, for example, from the setup of logistic elements adapted to families’ needs with training that allows for a good transition of life during the use of respite or from a stimulating environment tailored to and accepted by people with dementia [45, 49, 68, 70, 71, 77, 81–85].

The quality of care and the benefit of respite services are also major issues. Therefore, the assignment of a case manager, the generation of a protocol, time for the staff to recover, and the quality of the respite time are considered positive elements [58, 70, 71, 77, 86]. Continuity of care during the treatment process by a single caregiver and interventions that improve the transition from home to respite care promote the use of respite. For the quality of the care to be recognized, caregivers must be able to gauge the credibility and the legitimacy of the work the care staff carries out at home or in institutions.

##### The legitimacy afforded to the care staff

Confidence in patient care, recognition of professional abilities, and the legitimacy awarded to care teams promote the use of respite [48, 49, 51, 60, 61]. The care staff’s abilities were barriers and promoting factors. Indeed, caring for people with dementia requires specialized training to focus on the dignity of seniors, family values, ​​and relationships and to gain knowledge of the network specific to this population’s care. These elements are contributing factors to the use of respite services [41, 42, 58, 61, 68, 77, 83]. Care staff characteristics (e.g. confidence, honesty, etc.) have also been identified as positive elements [42]. A lack of follow-up, unawareness of caregivers’ needs, failure to consider the relationship between the dyad, and cultural differences between caregivers and carers are barriers to the use of respite [68, 70, 72, 76]. The care staff needs to gain legitimacy to work effectively with caregivers.

##### The partnership and respite’s impact

Caregivers’ lack of control is a barrier to the use of respite services. Indicators of a partnership, however, promote the use of respite interventions. Several factors comprise the existence of a positive relationship between the staff and the dyad: keeping a support diary to record the interventions the caregiver suggests, a way for the caregiver to retain a degree of control, the creation of a trusting relationship between the staff and the dyad, taking the caregiver’s knowledge into consideration, and effective communication between the staff and the caregiver [39, 51, 58, 68, 76, 83, 85]. The partnership between the care team and the caregiver helps promote the benefits of a respite break.

If the caregiver has been able to take advantage of respite time to perform a recreational activity and the patient does not decompensate or impose an increase in the caregiver’s workload upon their return, the caregiver will be renewed [42, 51, 74]. Indeed, when a caregiver has experienced respite, the decision to pursue this experience will depend on the respite period’s impact [74].

##### Caregivers’ abilities and identification of the need for respite

Caregivers need to develop cognitive abilities, a capacity for self-evaluation, and positive coping mechanisms that can support the transition to respite [49, 50, 55, 60, 61, 65, 87, 88]. To allow time for respite, the caregiver must also recognize the need for rest [49, 55, 88].

In summary, as indicated by the various themes that were highlighted, involvement and partnership with caregivers in the care of people with dementia is important. In many cases, caregivers strive to make their contributions by providing care, and it seems essential that they are fully integrated in the design of political and institutional projects. Indeed, the theme of respite service attributes was cited the most. If caregivers were integrated into projects, the proposed modalities and services would indirectly respond to the needs of caregivers and people with dementia.

#### Analysis of the recurrent themes

**Fig. 3:**
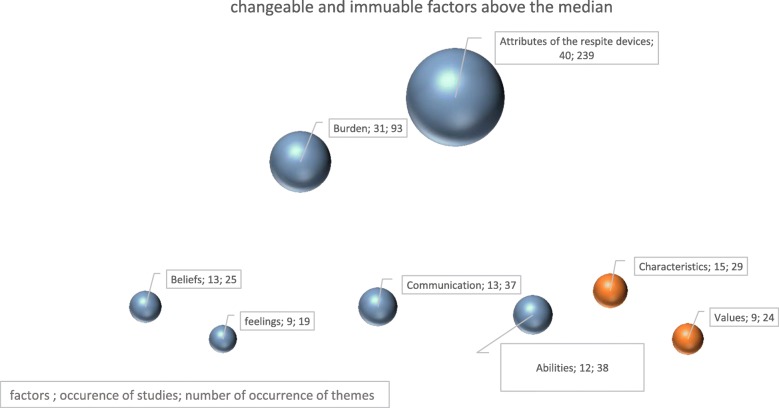
Chart of changeable and immutable factors

The classification of immutable and changeable factors makes it possible to better visualize the necessary actions to promote the use of respite. However, the numerous factors required a quantitative analysis to prioritize actions based on the literature’s most cited factors. The factors are ranked according to the median [median = 19], which allows the most cited—and factors to stand out. The elements taken into consideration for this classification are the number of studies who cited a particular factor and the number of times that it has been cited in the literature (see Fig. 3).

The main highlighted factors were “the attributes of the respite interventions” (cited 239 times by 40 studies) and “the burden” for caregivers (cited 93 times by 31 studies). These factors were followed by “communication” (cited 37 times by 13 studies) and, lastly, characteristics, beliefs, feelings, and values. The other factors are all referred to as “changeable.” Moreover, this analysis showed that more modifiable factors exist than immutable factors, which indicates possible change in favor of the use of respite services.

## Discussion

The purpose of this integrative review was to identify the factors that promote or hinder the use of respite interventions by caregivers for people with dementia. Several authors have already highlighted various elements, particularly regarding the problems of access to respite, the problems related to communication, and caregivers’ various expectations [30–32]. However, this study’s results show that the attributes of respite benefits are the factors that most cited in literature. By examining this factor in more detail, we have identified the notions of geographical distance, administrative complexity, and financial requirements as barriers to respite. O’Shea et al. (2017) and Phillipson et al. (2014) highlighted these elements in their systematic reviews, only geographical distance notion is a new element. On the other hand, our results highlight the need to change the way care is managed to foster networking and collaboration with caregivers. Participation in the network is essential to provide a multidisciplinary approach in the holistic management of the dyad. O’Shea et al. (2017) highlighted the importance of supporting the transition to the use of respite services. From that our study’s results, one can assume that this task could be assigned to a case manager. Indeed, several authors have supported the case manager’s importance in the coordination of complex care involving all care partners [71, 77], which ensures a personalized follow-up taking into account the dyad’s specific needs. Indeed, the use of respite services by caregivers of people with dementia is often low because the services do not seem to meet the caregivers’ expectations. Moreover, as noted in this study and in the systematic reviews by Neville et al. (2015) and O’Shea et al. (2017), communication problems arise between caregivers and respite care providers. The case manager could help reduce these problems. A partnership between health professionals and caregivers can help avoid communication problems and assimilate each of the two parties’ skills to potentiate a personalized procedure for the management of the dyad. Caregivers can guide decisions, for example, in developing a care plan. This is a new paradigm that health professionals still need to fully embrace. Indeed, this acceptance requires the will to share care [89]. This partnership also requires that the caregiver agrees to work with health professionals and thus use the respite services. This study’s results indicate that this small step is complicated for the caregiver. Indeed, Strang (2000) and Mollard (2009) indicated that notions of a cognitive nature, feelings, and values influence this decision [21, 74]. The partnership between health professionals and caregivers can minimize caregivers’ feelings of guilt, betrayal, insecurity, and loss of control and improve their quality of life [90]. To foster collaboration, health professionals must acquire self-assessment skills and support caregivers’ transitions to respite services [49, 55, 60, 61, 65, 87, 88]. However, it is important to recognize that experienced caregivers have, over the years of care, acquired substantial skills [91, 92]. Through their partnership, the use of their skills will allow for recognition of the caregiver’s role in society [9].

Based on the importance of respite services’ attributes, the benefits provided to the care recipient must be high-quality and personalized. Several authors indicated that to guarantee the quality of respite care, measures must be considered in institutional and home respite care facilities [30, 53].

This finding requires the involvement of policy makers and health professionals. This integrative review has highlighted “changeable” and “unchangeable” factors that influence the decision to access respite. Actions that may affect the “changeable” factors relate directly to health policies [93], particularly the decisions that guide the implementation of respite interventions. However, a large part of the responsibility is attributed directly to health professional, who by providing daily care have a pronounced influence on patients’ quality of life [94, 95]. The partnership with health staff can affect access to respite, patient-centered care, personalization of care according to the dyad’s needs, the sense of burden, the quality of the care provided, the beliefs of caregivers, and the credibility that they are afforded.

The immutable factors, that is to say ethnicity, values, the relationship that exists in the dyad, and the characteristics of caregivers and the individuals living with the disease, are not modifiable. It is policy makers’ and health professionals’ responsibility to adapt to these indicators and take them into consideration upon the creation or alteration of respite interventions and in the daily provision of care. These actions are important for addressing caregivers’ needs and expectations in terms of their role as carers, the intervention attributes caregivers expect, and their expectations regarding the respite’s effects on themselves and their relatives living with the disease. Unlike other types of systematic reviews, this review’s analysis method revealed the link between the various factors influencing the use of respite services and the people they affect. In addition, it highlights the fact that the use of caregivers’ skills in partnership with politicians, decision makers, and care teams can influence the use of respite services. Beyond the information caregivers learn when caring for people with dementia, they can provide solutions in the care of people with dementia, boosting their and their charges’ health and avoiding unnecessary hospitalizations that pose a considerable cost to society and to the safety and quality of care. Respite services must be redesigned to integrate caregivers’ experiential skills into care. A limitation of this study is that the majority of cases occurred in the United States. Due to the difference in ethnicity and health culture, this factor could hamper the transposition of results. In addition, the analysis corresponding to the prioritization of the factors cited in the literature has limitations. Indeed, the most cited factors may have more to do with the availability of data, the factor’s popularity, or the researchers’ biases than with the priority areas.

## Conclusion

This study’s results reveal important elements that policy makers and institutional decision makers should consider. The functioning and organization of respite services must meet the dyad’s specific needs. To meet caregivers’ needs, future research could focus on the evaluation of a systemic support program that could meet all the needs of caregivers. It should integrate the need for information, training, recognition, respite, socialization, and financial support. This intervention could be designed based on the fundamentals of a so-called win-win partnership.

## Additional files

Additional file 1: The search equations used were as follows for the articles. (DOCX 12 kb)

Additional file 2: MMAT grill evaluation. Reference: Pluye, P., Robert, E., Cargo, M., Bartlett, G., O’Cathain, A., Griffiths, F., Boardman, F., Gagnon, M.P., & Rousseau, M.C. (2011). Proposal: A mixed methods appraisal tool for systematic mixed studies reviews. Retrieved on [date] from http://mixedmethodsappraisaltoolpublic.pbworks.com. Archived by WebCite® at http://www.webcitation.org/5tTRTc9yJ. (DOCX 310 kb)

Additional file 3: summary table.docx. This table lists all selected articles by specifying the article reference, study type, purpose, participants, and MMAT quality score where indicated. (DOCX 64 kb)

## Abbreviations

MESH: Medical subject headings
MMAT: Mixed methods appraisal tool
