# Supplementary material for: Barriers and enablers in the use of respite interventions by caregivers of people with dementia: an integrative review
Source: Arch Public Health. 2018 Nov 22;76:72. doi: 10.1186/s13690-018-0316-y (PMC6249779; doi:10.1186/s13690-018-0316-y)
Supplement: Supplementary file 1 — The search equations used were as follows for the articles. (DOCX 12 kb) [file 13690_2018_316_MOESM1_ESM.docx]

Appendix 1 : The search equations used were as follows for the articles in English, in French, in Portuguese and in Spanish de 1980 à 2016.

| Data bases search | Research equation |
| --- | --- |
| PUBMED | (((caregivers AND ( ( "1980/01/31"[PDat] : "2016/12/31"[PDat] ) AND Humans[Mesh] AND ( English[lang] OR Portuguese[lang] OR French[lang] OR Spanish[lang] ) ))) AND ((((dementia AND ( ( "1980/01/31"[PDat] : "2016/12/31"[PDat] ) AND Humans[Mesh] AND ( English[lang] OR Portuguese[lang] OR French[lang] OR Spanish[lang] ) ))) OR (alzheimer AND ( ( "1980/01/31"[PDat] : "2016/12/31"[PDat] ) AND Humans[Mesh] AND ( English[lang] OR Portuguese[lang] OR French[lang] OR Spanish[lang] ) ))) AND ( ( "1980/01/31"[PDat] : "2016/12/31"[PDat] ) AND Humans[Mesh] AND ( English[lang] OR Portuguese[lang] OR French[lang] OR Spanish[lang] ) ))) AND (respite AND ( ( "1980/01/31"[PDat] : "2016/12/31"[PDat] ) AND Humans[Mesh] AND ( English[lang] OR Portuguese[lang] OR French[lang] OR Spanish[lang] ) )) |
| CINALH | (((MH "Caregivers") OR "caregivers" OR (MH "Caregiver Burden") OR (MH "Caregiver Support") OR (MH "Risk for Caregiver Role Strain (NANDA)") OR (MH "Caregiver Role Strain (Saba CCC)") OR (MH "Family Caregiver Status (Iowa NOC)") OR (MH "Caregiving Endurance Potential (Iowa NOC)") OR (MH "Caregiver-Patient Relationship (Iowa NOC)") OR (MH "Caregiver Well-Being (Iowa NOC)") OR (MH "Caregiver Support (Iowa NIC)") OR (MH "Caregiver Stressors (Iowa NOC)") AND((MH "Alzheimer's Disease") OR "alzheimer") OR ((MH "Dementia") OR "dementia" OR (MH "Frontotemporal Dementia") OR (MH "Dementia, Vascular") OR (MH "Delirium, Dementia, Amnestic, Cognitive Disorders") OR (MH "Dementia, Multi-Infarct") OR (MH "AIDS Dementia Complex") OR (MH "Lewy Body Disease") OR (MH "Dementia, Senile") OR (MH "Dementia, Presenile") OR (MH "CADASIL"))) AND ((MH "Respite Care (Iowa NIC)") OR (MH "Respite Care") OR "respite") |
