# Supplementary material for: Barriers and enablers in the use of respite interventions by caregivers of people with dementia: an integrative review
Source: Arch Public Health. 2018 Nov 22;76:72. doi: 10.1186/s13690-018-0316-y (PMC6249779; doi:10.1186/s13690-018-0316-y)

Appendix 2 : MMAT grill evaluation, reference : Pluye, P., Robert, E., Cargo, M., Bartlett, G., O’Cathain, A., Griffiths, F., Boardman, F., Gagnon, M.P., & Rousseau, M.C. (2011). *Proposal: A mixed methods appraisal tool for systematic mixed studies*

*reviews.* Retrieved on [date] from http://mixedmethodsappraisaltoolpublic.pbworks.com. Archived by WebCite® at http://www.webcitation.org/5tTRTc9yJ


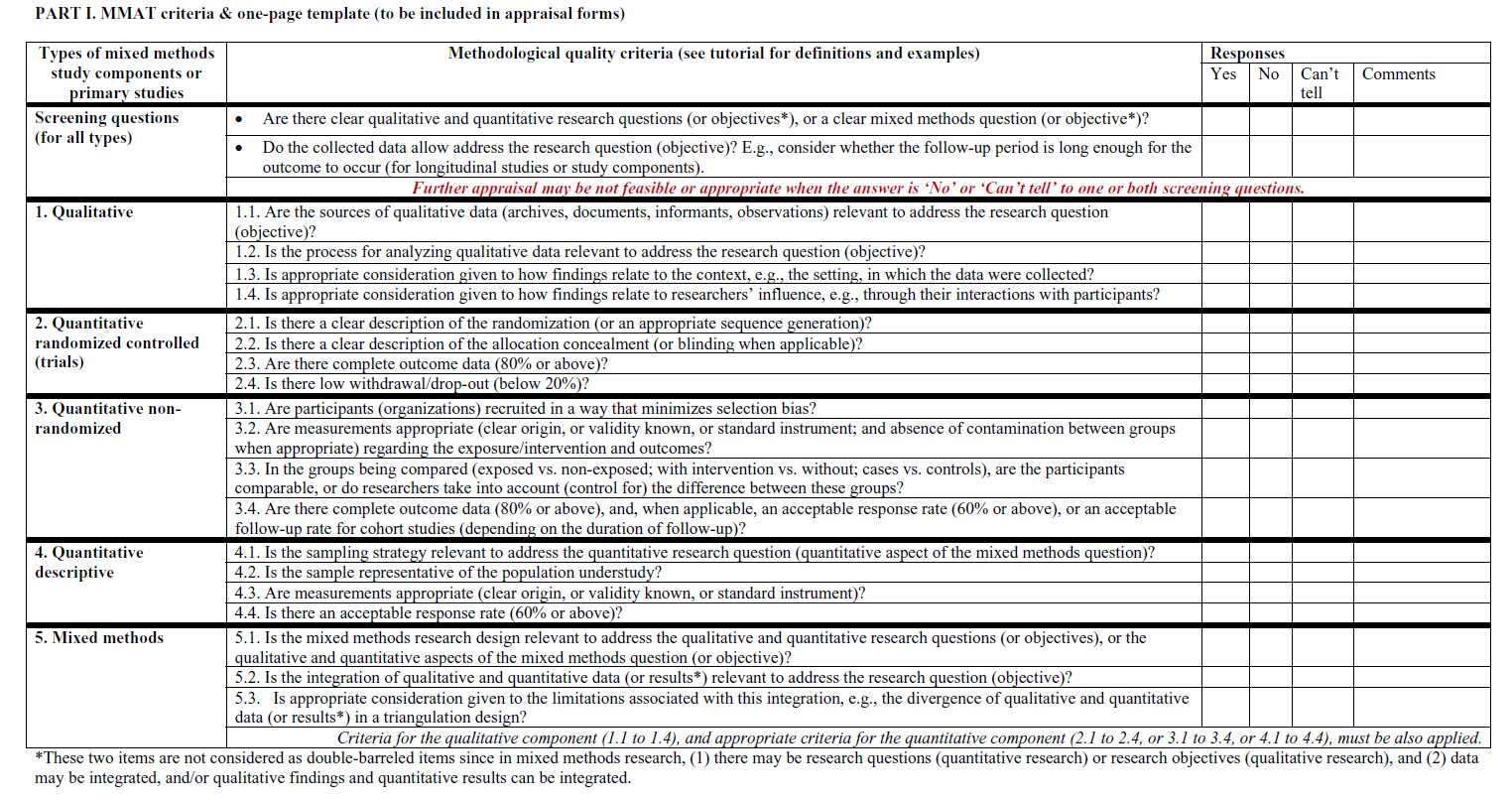

Supplement: Supplementary file 2 — MMAT grill evaluation. Reference: Pluye, P., Robert, E., Cargo, M., Bartlett, G., O’Cathain, A., Griffiths, F., Boardman, F., Gagnon, M.P., & Rousseau, M.C. (2011). Proposal: A mixed methods appraisal tool for systematic mixed studies reviews. Retrieved on [date] from http://mixedmethodsappraisaltoolpublic.pbworks.com. Archived by WebCite® at http://www.webcitation.org/5tTRTc9yJ. (DOCX 310 kb) [file 13690_2018_316_MOESM2_ESM.docx]
