# Supplementary material for: Barriers and enablers in the use of respite interventions by caregivers of people with dementia: an integrative review
Source: Arch Public Health. 2018 Nov 22;76:72. doi: 10.1186/s13690-018-0316-y (PMC6249779; doi:10.1186/s13690-018-0316-y)
Supplement: Supplementary file 3 — summary table.docx. This table lists all selected articles by specifying the article reference, study type, purpose, participants, and MMAT quality score where indicated. (DOCX 64 kb) [file 13690_2018_316_MOESM3_ESM.docx]

Appendix 3 : References of studies and selected documents

| Reference | Article type | Aim | Participant | Quality Score MMAT^[[1]](#footnote-1)^ | Country | The year of study | The follow-up period | Details of intervention. |
| --- | --- | --- | --- | --- | --- | --- | --- | --- |
| Adler, G., Kuskowski, MA., Mortimer, J. (1995) Respite use in dementia patients. Clinical Gerontologist, 15(3), 17-30. | Quantitative descriptive | "To predict who will request respite care and to identify the impact attitudes may have on its use". | 58 caregivers of dementia patients treated in an outpatient memory loss clinic | MMAT = 4/4 | USA  (New-York, New Orleans) | 1993 | Paticipants were tracked for one year after the study began | **Method :**  «The sample for this study was comprised of caregivers of demented individuals who received ongoing care at a memory loss clinic at the Minneapolis Veterans Affairs Medical Center (VAMC). Care providers were initially contacted by telephone and invited to participate at the study.All familiy treated by the memory loss clinic received information by letter of the availability of institutional respire care (they could arrange for one to two week stays quartly with a maximum of four weeks annually) » and « a one or two week free institutional respite program at hospital was proposed »  **Type of respite studed:**  « Formal community service use was measured by asking caregivers if they were currently using the following : support groups, visiting nurses, adlut day care, family therapy, home health aides, transportation services, financial counseling, case management, meal programs, senior companion and other services » |
| Bakker, C., de Vugt, M., Vernooij-Dassen, M., Van Vliet, D., Verhey, FR., Koopmans, RT. (2010) Needs in early onset dementia: A qualitative case from the NeedYD study. American Journal of Alzheimers Disease & Other Dementia, 25(8),634-40. doi: 10.1177/1533317510385811. PMID:21131669 | Qualitative case study | "To explore the experiences of a caregiver of a patient with early onset dementia and the needs of patient and caregiver". | A single case study | MMAT= 3/4 | Netherlands | 2010 | data were collected at time of inclusion and after 6 and 12 months | **Method :**  **«**Qualitative interviews with the caregiver were used to explore how the caregiver perceived met and unmet needs of both the patient and caregiver and how she experienced transitions in care and health care services provided.  **Type of respite studed :**  **«**support groupes, daycare facilities and respite care » |
| Beeber, AS.,Thorpe, JM., Clipp, EC. (2008) Community-based service use by elders with dementia and their caregivers: a latent class analysis. Nursing Research,;57(5),312-21. doi: 10.1097/01.NNR.0000313500.07475.eb. PMID:18794715 | Quantitative non-randomized (cross-sectionnal analytic study) | "To classify care recipients with dementia and their caregivers who shared similar patterns of CBSS use and to identify predictors of class membership". | 1813 participants | MMAT= 4/4 | USA  (United States and Puerto Rico) | 2008 | Dataset from 1998 | **Method :**  «This study was a cross-sectional secondary analysis of the  National Longitudinal Caregiver Study (NLCS) dataset using  latent class analysis (LCA) to illustrate patterns of CBSS use »  **Type of respite studed : «**caregiver support group membership,home health, in-home aide, home health, adult day care and respite care » |
| Biegel, DE., Bass, DM., Schulz, R., Morycz, R.(1993). Predictors of in-home and out-of-home service use by family caregivers of Alzheimer's disease patients. *Journal of Aging an*d Health. ,5(4), 419-38. PMID:10171716 | Quantitative descriptive | A comparaison of 4 groupes of elderly care recipients. | 171 participants | MMAT= 4/4 | USA  (Pittsburgh, Cleveland) | 1993 | A classification was made 6 month preceding the interviews | **Method :**  « Interviews of family members of a university-affiliated Alzheimer’s disease diagnostic centers, were conducted in the caregiver’s home and took approximately 90 minutes. Families were classified into four groups based on the patient’s use of services : (a) users of in-home service only ; (b) users of out-of-home service only ; (c) users of both in-home and out-of-home services ; and (d) those using neither in-nor out-of-home services.This classification reflects service contact or whether or not services were used, regardless of the volume, duration, or number of different services used. They used the ADL measure. »  **Type of respite studed :**  « in-home services : home health aide services, housekeeping, nursing care, delivered meals. Out-of-home services : adult day care, senior center meals and transportation. |
| Bigony, MD.(2007) Perceptions of the nurse-caregiver relationship and its influence on the utilization of respite care services by spousal caregivers of patients diagnosed with dementia. *Catholic University of America*, 2007 PH.D. 11 p-111. | Thesis | Perceptions of the nurse-caregiver relationship and its influence on the utilization of respite care services by spousal caregivers of patients diagnosed with dementia. | 15 participants | Level of Evidence = 4 | USA  (New-York,  Washington DC) | 2007 | No following period | **Method :**  « Interviews about caregivers perceptions of the relationship with nurses»  **Type of respite studed :**  « In-home services, adult day care, nursing home or hospital » |
| Blume, L., Persily, NA., Mirones, M., Swaby-Thorne, A., Albury, S. (1990) Anatomy of the Alzheimer's Respite Care Program (ARCP) *Home Health Care Services Quarterly*, 11 (3/4), 75-90. | Expert opinion | Comparaison between the original project conceptualization with the current design today. |  | Level of evidence = 6 | USA  (New-York, Florida) | 1990 | The project lasted seven months | **Subject discussed:**  « Expand the Alzheimer respite care program by « development of program brochure ; advertisement in local newspapers ; coverage on local television stations ; publicity visits with community agencies and hospitals ; and marketing internally to the project’s parent organizations »  **Type of respite studed :**  « in-home service, short term institutional respite, institutional respite for vacations or medical emergencies and in-service training » |
| Butterworth, M. (1995) Dementia: the family caregiver's perspective. *Journal of Mental Health*, 4(2), 125-132. | Expert opinion | From account of a daughter who has been for fifteen years and still is caring for her mother given a conference held in London, conclusions for service was draw. |  | Level of evidence = 6 | UK  (London) | 1995 | No following period | **Subject discussed :**  « The personal account of a daughter is presented, who, over a period of fifteen years, has cared for her mother with multi-infarct dementia at home. The information to give at caregivers, the admiral nurse service, problems encountered, the cost, training, dementia specific care are also discussed »  **Type of respite studed :**  « social services, home care, day centre, local home respite, local hospital respite » |
| Clark, M., Bond, MJ. (2000) The effect on lifestyle activities of caring for a person with dementia. *Psychology, Health & Medicine*, 5(1), 13-27 . | Quantitative descriptive | Examined the participation in lifestyle activities of spouse caregivers of people with dementia. | 163 couples | MMAT = 3/4 | Australia | 2000 | « Caregivers were asked to indicate how often in the last three months they had used respite » | **Method :**  « a cross-sectional comparison of three groups of couples defined according to the progression of the dementia The data were analysed with four instruments : Adelaide Activites Profile, Geriatric Depression Scale, SF-36 Health Survey and Functional Dementia Scale »  **Type of respite studed :**  « day respite services such as day care centres, activity centres and respite service such as hostels or nursing homes» |
| Clark, M.(2011) A little respite: essential bundle. *Perspective infirmière*, 8(4), 16-16. | Expert opinion | Presentation and story of service "Baluchon Alzheimer". |  | Level of evidence = 6 | Canada (Québec) | 2011 | No following period | **Subject disscused :**  « Many caregivers preferred not to take a break rather than entrust their relatives with Alzheimer's disease at a respite home, as it was often painful and the caregivers were disappointed. Marie Gendron, a nurse at the Research Center, spontaneously offered to help these caregivers replace them.  **Type of respite studed :** « Respite and home support service for caregivers of people with Alzheimer's disease » |
| Cotrell, V. (1997) Respite Use of Dementia Caregivers. *Journal of Gerontological Social Work*, 26:3-4, 35-55. DOI: 10.1300/J083V26N03 04 | Quantitative descriptive | For each form of respite , caregivers were asked to respond ton and amplify their views on two questions: Explain how each respite (sitter, daycare, and overnight) might be used in meeting your present or future caregiving needs, and describe the factors or events that led to your initial use of the service. | 100 participants | MMAT = 3/4 | USA  (Texas) | 1997 | No following period | **Method :**  « personal interviews in the homes of caregivers »  **Type of respite studed :**  « in-home sitter service, home health agencies, nursing homes, daycare facility which had overnight capacity » |
| Cotrell, V., Engel, RJ.(1999) The Role of Secondary Supports in Mediating Formal Services to Dementia Caregivers. *Journal of Gerontological Social Work*, 30(3/4), 117-132. | Quantitative descriptive | "To provides a preliminary investigation of the mediative function of professionnal and informal supports as it relates to the use of 3 types of respite services by dementia caregivers". | 100 participants | MMAT = 3/4 | USA  (Texas) | 1999 | Not mentioned | **Method :**  Personal interwiews with 100 primary caregivers who were of the Alzheimer’s Association  **Type of respite studed :**  « in-home sitter service, home health agencies, nursing homes, daycare facility which had overnight capacity » |
| Cox, C. (1997) Findings from a Statewide Program of Respite Care: A Comparaison of Service Users, Stoppers, and Nonusers. *Gerontologist*, 37(4), 511-517. PMID: 9279040 | Quantitative descriptive | Compared respite users and non users in the Health Resources and Services Administration-funded Alzheimer's disease demonstration grant in the State Administration of Maryland. | 228 participants | MMAT = 3/4 | USA  (Maryland) | 1997 | Interviews were conduct in 1992 and 1993. The interviews were conducted 6 months after having the sample of the population | **Method :**  « interviews with 228 caregivers accepted into Maryland’s Alzheimers »  **Type of respite studed :**  « inhome care, short stays in a nursing home, day care. |
| DeCaporale, L., Mensie, L., Steffen, A. (2013) Respite utilization and responses to loss among family caregivers: relationship matters. Death Studies. 37(5),483-92. PMID:24517567 DOI: 10.1080/07481187.2012.654593 | Quantitative descriptive | "To examine the longitudinal relationship between grief reaction in current spousal and adult-children caregivers & in-home respite utilization over 3 months". | 72 participants | MMAT= 3/4 | USA  (New-York,  Missouri) | 2013 | 3 months after the telephone | **Method :**  « a 90 minutes telephone interview with familiy caregivers registered in-home respite »  **Type of respite studed :**  « in-home respite services » |
| De la Cuesta-Benjumea, C. (2010) The legitimacy of rest: conditions for the relief of burden in advanced dementia care-giving. *Journal of Advanced Nursing.* 66(5), 988-998. PMID: 20337791 | Qualitative grounded theory | "To identify the condition that favour the relief of the burden of female caregivers of relatives with advanced dementia". | 22 participants | MMAT = 4/4 | Spain  (Alicante) | 2010 | The interviews were conduced between november 2006 and may 2008 | **Method :**  « 22 semi-structured interviews lasted 40-90 minutes, were audio taped and transcribed verbatim »  **Type of respite studed :**  « Informal respite : caregivers searched for substitutes among cohabiting relatives, such as spouses, parents and offspring » |
| Ducharme, F., Lévesque, L., Ethier, S. Lachance, L. (2007) Masculine' care: older husband caregivers' perceptions of the caregiver experience and services. *Canadian Journal of Community Mental Health*, 26(1), 143-159. | Qualitative phenomenological study | Comparison of two groups of men in their experience of helping their spouse & their perception of services. | 43 participants | MMAT= 3/4 | Canada  (Monréal, Québec, Sherbrooke et Saguenay-Lay-St-Jean) | 2007 | No following period | **Method :**  « 45 to 60-minute home interview of caregivers who participated in a longitudinal study a year earlier »  **Type of respite studed :**  « Local Community Service Centers, Alzheimer Societies,day care centers, external cognition clinics » |
| Ehrlich, P., White, J.(1991). TOPS: a consumer approach to Alzheimer's respite programs. *Gerontologist*. 31(5),686-91.PMID: 1778496 | Expert opinion | A program for caregivers of Alzheimer's victims, provides in-home and day program services. |  | Level of evidence = 6 | USA  (Cleveland) | 1991 | Follow-up visit two months after the program begin | **Subject discussed** :  « The caregivers received a follow-up visit by professional staff. The  worker may provide limited (two visits) education or supportive counseling. A follow-up monitoring visit schedule is then determined on a case-by-case basis, and regular telephone contact with the caregiver is  maintained »  **Type of respite studed :**  « in-home respite aide service, day care centres, overnight service, support systems, homemaker-personal care service » |
| Galvin, JE., Duda, JE., Kaufer, DI., Lippa, CF., Taylor, A., Zarit, SH. (2010) Lewy body dementia: caregiver burden and unmet needs. Alzheimer's Disease Association Disorder, 24(2), 177-81. doi: 10.1097/WAD.0b013e3181c72b5d. PMID:  20505434 PMCID:  PMC2879080 | Quantitative descriptive | Survey was to ascertain the unmet needs of LBD caregivers and collect date to inform educationnal and enhance caregiver support. | 971 participants | MMAT= 3/4 | USA (Washington) | 2010 | The survey was posted on the LBDA website  for six months | **Method :**  « An internet-based survey was developed by the LBDA and placed on-line using Survey  Monkey »  **Type of respite studed :**  « in home assistance, adult day care, support group » |
| Gendron, M., Adam, E. (2005) Caregiving challenges. Baluchon Alzheimer©: an innovative respite and support in the home of the family caregiver of a person with Alzheimer's. *Alzheimer's Care Quarterly*, 6(3), 249-261. | Qualitative phenomenological study | Description of a new respite and support service, Baluchon Alzheimer, concerning the respite part of the service based on the behavioral model of families' use of health services. | 14 participants | MMAT= 3/4 | Canada  (Nova Scotia) | 2005 | One year after the start of the intervention | **Method :**  « a pre-intervention research was effectued few day before the intervention and then the caregiver answered a post-intervention research questionnaire. Some caregivers requested the service a second time for purposes of comparison they asked to complete a second post-intervention research questionnaire. After one year a focus group was effectued  **Type of respite studed :**  «  home care service of respite and support » |
| Gwyther, LP.(1989).Overcoming barriers. Home care for dementia patients. *Caring,* 8(8):12-6. PMID:10318309 | Expert opinion | Highlight a situation in which a dementia-capable and dementia-specific in-home respite service was available, and identifiy factors that may have interfered with its timely and effective use. |  | Level of evidence = 6 | USA  (North Carolina, Durham) | 1989 | Two year study period | **Subject discussed**:  « The Duke In-Home-Respite Care Model : Two urban counties were served by a traditional proprietary home health agency. Traditional long-term care agencies were chosen. The services were available at scheduled times, but flexible night and weekend hours. Subsides were available up to 40 dollars per week for up to 20 families. All respite care provider were nursing assistants with one year of experience in either a nursing home or home health setting and trained by Duke faculty »  **Type of respite studed :**  «  in-home respite » |
| Ham, RJ.(1999) Evolving standards in patient and caregiver support. *Alzheimer Disease and Associated Disorders*. 13 Suppl 2, S27-35. | Expert opinion | « Role of the caregiver in Alzheimer disease management ». |  | Level of evidence = 6 | USA  (New-York) | 1999 | No following period | **Subject discussed**: « Strive to educate and train the caregiver to be as effective as possible in their caregiving role. Promote a more positive and proactive mind set, focusing ont he caregivers often untapped potential to become a skilled member of therapeutic team »  **Type of respite studed :**  « respite services, day care, counseling and support groups » |
| Hayes, JM. (1999) Respite for caregivers: a community-based model in a rural setting. *Journal of Gerontological Nursing*, 22-53. PMID: 10205420 | Expert opinion | A model of respite services is proposed to provide community-based respite services in a rural setting in northeast Georgia. |  | Level of evidence = 6 | USA  (Northeast Georgia) | 1999 | 2 years | **Subject discussed :**  « The Community-Based Model : flexible regarding the activities and services provided. To ease the concerns of caregivers. Included assessment of client and caregiver needs, caregiver education, case management, careful monitoring of clients, counseling for caregivers ans transportation. Additional services include caregiver counseling in the form of support group activites, education, and guidance by program staff »  **Type of respite studed :**  « in a senior center who prupose : home-delivered meals, homemaking services and congregate activites » |
| Koffman, J,. Taylor, SJC. (1997) The needs of caregivers…service review. *Elderly Care*, 9(6), 16-19. | Qualitative phenomenological study | Exploring respite care for caregivers and their dependants with dementia inner London health and what caregivers really think about the service available. | A focus group with informal caregivers | MMAT= 3/4 | UK  Great Britain,  London) | 1997 | Not mentioned | **Method :**  « the study employed a focus group in order to elicit informal caregivers’perspectives of their role and experiences of caring, their views on local respite services, and to determinate what they considered was required of existing service provision. The group’s discussion was tape-recorded and later transcribed. After discussion resulting from the focus group, the scritp was independently coded and later agreed on the identification of four common themes : « the maning of caring », « experiences of caring », « perceptions of respite services » and « caregivers’needs ».»  **Type of respite studed :**  « residential care, day care and day and night sitting relief services » |
| Kosloski, K., Schaefer , JP., Allwardt , D., Montgomery, RJV., Karner, TX (2002) The role of cultural factors on client's attitudes toward caregiving, perceptions of service delivery, and service utilization. *Home Health Care Services Quarterly*,21(3/4), 65-88. PMID: 12665072 | Quantitative descriptive | "To gain a better understanding of the role of culture in the use of respite services". | 315 participants | MMAT = 4/4 | USA  (Florida, Maine, Michigan, North Carolina, South Carolina, Washington) | 2002 | Not mentioned | **Method :**  Telephone interviews were conducted with a culturally diverse sample  of 315 caregivers of Alzheimer’s patients participating in the Alzheimer’s Disease Demonstration Grants to States (ADDGS) program.  Two sets of analyses were conducted.  The first set of analyses examined the relationships between the three cultural variables (ethnicity, relationship, and geographic location)  and two sets of client attitudes. The second set of analyses assessed  the links between respite use and caregiving attitudes and beliefs, and caregivers’ judgments about aspects of service delivery.  **Type of respite studed :**  « in-home respite, adult day care » |
| Lawton, MP., Brody, EM., Saperstein, A., Grimes, M. (1989) Respite services for caregivers: research findings for service planning. *Home Health Care Services Quarterly*, 10(1/2),5-32. | Quantitative randomized experimental trial | Evaluating of respite project for caregivers. | 315 participants | MMAT = 4/4 | USA  (Philadelphia) | 1989 | One year | **Method :**  « The respite services were procured from the Philadelphia Geriatric Center (PGC) and other existing local services wherever possible and consisted of three types. First, in-home respite services usually involved a sitter, a person to perform homemaker services, or one who provided personal care. All such services had in common the fact of spending a period of time in the household, during which the primary caregiver was free to do something else. A second type of respite service was adult day care and the third was institutional respite care, limited by our definition to a stay of no more than 21 days.  **Type of respite studed :**  « In-home respite, adult day care and institutional respite care ». |
| Lobb, MO. (1992) Barriers to respite use: factors influencing use and non-use. *Georgia State University*, (Doctoral Dissertation - research) | Thesis | "To identify demographic, social, and health-related factors predictive of in-home respite use versus non-use among family caregivers of a relative with Alzheimer's disease or a related disorder."To investigate perceived barriers to respite use among caregivers who were non-uisers of respite". | 77 participants | Level of Evidence = 4 | USA  (Atlanta, Georgia) | 1992 | Not mentioned | **Method :**  « In order to identify subjects for the study, a group of 18 state affiliated Alzheimer’s service programs were located which provided in-home respite, out-home respite, and/or adult day-care services depending on the needs of the caregivers in their community. Fifteen of the 18 programs provided in-home respite services ».  **Type of respite studed :**  « In-home respite, out-home respite and adult day care service »- |
| Longshaw, S., Perks, A.(2000) Practice development. Respite care innovations for carers of people with dementia. *British Journal of Nursing*, 9(16), 1079-1081. PMID: 12785088 | Expert opinion | "To investigates carer belief regarding out-home respite serviced and why some carers do not utilise them". |  | Level of evidence = 6 | UK  (Sheffield) | 2000 | No following period | **Subject discussed:**  « In addition to existing traditional models of respite provision, a new service should be introduced : The Community Dementia Support Service. The service would operate between 7.00 am and 9.00 pm, 7 days a week, 365 days a year and would be open to carers of people with a confirmed diagnosis of dementia. The service provided is totally flexible and available for between half an hour to 12 hours per week, with current funding allowing  for a total of 150 care hours per week »  **Type of respite studed :**  « In-home service and Out-of-home service ». |
| Lucet, F. (2015) In-home respite for the families of Alzheimer's patients. *Soins Gerontologie,* (115),24-9. PMID:26364818 DOI:10.1016/j.sger.2015.07.006 | Expert opinion | The balluchonage as an innovative and unique model of respite and home support for people with Alzheimer's disease and their caregivers. |  | Level of evidence = 6 | Canada  (Québec)  Belgique | 2015 | No following period | **Subject discussed :**  « former caregiver, trained and accompanied by the Baluchon Alzheimer who offers a 24-hour home accompaniment and a respite duration ranging from 4 to 14 days »  **Type of respite studed :**  « in-home respite aide service » |
| MaloneBeach, EE., Zarit, SH., Spore, DL.(1992). Caregivers' perceptions of case management and community-based services: barriers to service use. *The Journal of Applied Gerontology,* 11(2),146-59. PMID:10171017 | Quantitative descriptive | "To explored the use of case-management services for dementia patients and their families". | 46 participants | MMAT= 3/4 | USA  (Pennsylvania) | 1992 | Not mentioned | **Method :**  «  a statewide program of case management operated through local aera agencies on aging. The publicly funded programs provide and broker services for frail elders living within the region of Pennsylvania. »  **Type of respite studed :**  « support groups, nursing home, board-and-care facility » |
| McGrath, WL., Meuller, MM., Brown, C., Teilelman, J., Watts, J. (2000) Caregivers of persons with Alzheimer's disease: an exploratory study of occupational performance and respite. *Physical & Occupational Therapy in Geriatrics*, 18(2), 51-69. | Qualitative phenomenological study | Explored the impact of caring for a family member with Alzheimer's on the caregivers occupational performance and the perceived influence of respite on occupational performance. | 5 participants | MMAT= 3/4 | USA  (Virginia) | 2000 | Not mentioned | **Method :**  « an extensive semi-structured interview was chosen. A qualitative, phenomenological framework was used in order to permit flexibility in the examination of the  research questions. In addition, an emergent research design allowed  the interview process to evolve from the collected and analyzed data »  **Type of respite studed :**  « home health, adult daycare » |
| Montgomery, RJV., Marquis, J., Schaefer, JP., Kosloski, K .(2002) Profiles of respite use. *Home Health Care Services Quaterly*, 21(3/4), 33-63. PMID: 12665071 | Quantitative descriptive | To identify and document long-term profiles of respite use among a diverse sample of families caring for elders with dementia. | 2395 participants | MMAT = 4/4 | USA  (Columbia, Florida, Maine, Michigan, North Caroline, South Caroline and Washington | 2002 | Between September 1992 ans December 1998 | **Method :**  « structured data collection instrument was mailed or faxed to the state coordinator of the ADDGS sites and to agency service providers to obtain programmatic information pertaining to the 112 agencies providing day care and in-home services. Telephone interviews were conducted with all respondents who did not return the form within two  weeks. In all cases, follow-up telephone calls were made to clarify any  information that was not completely reported »  **Type of respite studed :**  « in-home respite or day care services » |
| Montoro-Rodriguez, J., Kosloski, K., Montgomery, RJ. (2003) Evaluating a practice-oriented service model to increase the use of respite services among minorities and rural caregivers. *Gerontologist*, 43(6),916-24. PMID:  14704392 | Quantitative descriptive | To evaluate the practice-oriented model of service use relative to the more widely used behavioral model in its ability to explain the use of respite services by caregivers of Alzheimer's patientes. | 1158 participants | MMAT= 4/4 | USA  (South Carolina, Washington, DC, Maine, Puerto Rico, Michigan, Ohio, Florida,  Maryland, Montana,  California, Washington, North Carolina, and Hawaii) | 2003 | Not mentioned | **Method :**  « Data were collected in 1996 through telephone  interviews with 1,183 caregivers. A structured interview protocol was used to elicit caregivers’  attitudes about their use of respite services »  **Type of respite studed :**  « adult day care, in-home respite » |
| Neville, CC., Byrne, GJA. (2002) Behaviour of older people admitted for residential respite care. *Australian Journal of Advanced Nursing*, 20(1), 8-12. PMID: 12405277 | Quantitative descriptive | To determine whether residential respite is used because of disruptive behavior displayed by older people. | 35 participants | MMAT = 4/4 | Australia  (Queensland) | 2002 | the study was conducted over a three-month period. | **Method :**  « Data were obtained  by the nurses from the respite recipients’ clinical records  **Type of respite studed :**  « residential respite » |
| O'Connell, B., Hawkins, M., Ostaszkiewicz, J., Millar, L. (2012). Carers' perspectives of respite care in Australia : An evaluative study. *Contemporary Nurse*. 41 (1), 111-119. PMID : 22724912 | Quantitative descriptive | To examinate carers experiences of using all types of respite care available and their views. | 62 participants | MMAT = 4/4 | Australia  (Melbourne) | 2012 | Not mentioned | **Method :**  « This descriptive study used a self-report, replypaid mailed survey package that was distributed to  approximately 300 carers of people with dementia »  **Type of respite studed :**  « regular outings, day centre, residential care facilities, overnight care in a house or cottage |
| Perry, J., Bontinen, K.(2001) Evaluation of a weekend respite program for persons with Alzheimer disease. *Canadian Journal of Nursing Research*, 33(1), 81-95. PMID: 11928159 | Qualitative phenomenological study | An exploration of the experiences of family caregivers in a pilot moral support program that provides weekend care for people with Alzheimer's disease or related dementia. | 18 participants | MMAT = 3/4 | Canada | 2001 | The caregivers used the service over 6 month period | **Method :**  « an Adult Day Program (ADP) : he ADP operates as an eight-bed overnight respite service from Friday afternoon to Monday morning. Family caregivers completed a care booklet containing information on the habits and preferences of the person with dementia. The program is designed to give clients the freedom to maintain personal schedules and routines »  **Type of respite studed :**  «  an adult day program ». |
| Phillipson, L., Jones, SC. (2011) Residential respite care: the caregiver's last resort. *Journal of Gerontological Social Work*, 54(7),691-711. doi: 10.1080/01634372.2011.593613. PMID:21967139 | Qualitative phenomenological study | To improve understanding of the beliefs that may influence the use and non-use of RRC by caregivers of people with dementia via the application of theory. | 36 participants | MMAT= 3/4 | Australia | 2011 | Not mentioned | **Method :**  « Recruitment was via letter through an agency coordinating the local branch of a national respite telephone call center, and caregivers were subsequently followed up by  Phone. Respondents were provided with a choice of participation in individual interviews, dyadic interviews, and focus groups »  **Type of respite studed :**  « nursing home, aged care hostel, or respite cottage » |
| Phillipson, L., Jones, SC. (2012) Use of day centers for respite by help-seeking caregivers of individuals with dementia. *Journal of Gerontological Nursing*, 38(4),24-34. quiz 36-7. Doi: 10.3928/00989134-20120307-05. PMID:22420521 | Qualitative phenomenological study | Utilises theory to conceptualise the behavioral, normative and control beliefs that caregivers of people with dementia associate with the use of home day centers of respite. | 36 participants | MMAT= 4/4 | Australia | 2012 | Not mentioned | **Method :**  « Recruitment was via letter through an agency coordinating the local branch of a National  Respite Telephone Helpline. Caregivers were followed up by telephone. Data were collected utilising  focus groups, individual interviews and interviews dyads»  **Type of respite studed :**  « out of home day centers » |
| Phillipson, L., Magee, C., C. Jones, S. (2013) Why carers of people with dementia do not utlise out-of-home respite services. *Health & Social Care in the Community*, 21(4), 411-422. PMID: 23496258 | Quantitative descriptive | To investigates carer belief regarding out-home respite serviced and why some carers do not utilise them. | 152 participants | MMAT = 3/4 | Australia | 2013 | Between November 2008 and  November 2009 | **Method :**  **«**Carers who had made contact with the two  Commonwealth funded respite assessment and  approval services in the lllawarra region were mailed a Research Pack (an information sheet, questionnaire, consent form and a reply paid envelope). In addition, local community  and health services and the Alzheimer’s NSW  Telephone Helpline provided eligible carers with Research Packs by mail or in person and advertisements for the study were also placed in the Alzheimer’s NSW supporters’magazine »  **Type of respite studed :**  « day care, residential services » |
| Robinson, K, M., Buckwalter, K., Reed, D. (2013). Differences Between Dementia Caregivers Who are Users and Nonusers of Community Services. *Public Health Nursing,* 30 (6), 501-510. PMID : 24579710 | Quantitative non-randomized (cross-sectionnal analytic study) | To examine differences between users and non-users of community services in caregivers of persons with dementia. | 241 participants | MMAT = 3/4 | USA  (North Carolina) | 2013 | Data were collected between 1995 and 1997 and then from 2005 to 2007 and 2007 to 2010 | **Method :**  « The primary date were obtained from a  multi-site project using eight research sites. Data reported  in this manuscript were subsequently validated/supported  by two subsequent studies on different community »  **Type of respite studed :**  « all community services » |
| Robinson, KM., Buckwalter, KC., Reed, D. (2005) Predictors of use of services among dementia caregivers…including commentary by Forbes with author response. *Western Journal of Nursing Research*, 27(2), 126-147. PMID: 15695566 | Quantitative descriptive | To examine predictors of use of community resources among caregivers of persons with dementia. | 241 participants | MMAT 3/4 | USA  (Iowa) | 2005 | 1-year data collection period | **Method :**  « The data for the current study were taken from the baseline assessments  of participants in a multisite experiment that used repeated measures. Six instruments were used to measure severity of disease, social support, caregiver burden, problem behavior, and depression »  **Type of respite studed :**  « respite services or  caregiving assistance, whether from professionals or nonprofessionals |
| Robinson, A., Lea, E., Hemmings, L., Vosper, G., McCann, D., Weeding, F. (2012) Seeking respite: issues around the use of day respite care for the carers of people with dementia; *Ageing & Society,* 32(2), 196-218. | Qualitative phenomenological study | To identified issues around the use of day respite care from the perspective of the family carer, focusing on barriers to attendance and strategies to facilitate attendance. | 27 participants | MMAT = 4/4 | Australia  (Tasmania) | 2012 | The study was conducted between August and December 2007 | **Method :**  « Twenty-seven telephone interviews were conducted : ten with carers whose family member refused to attend day respite care and 17 with carers whose family member attended day respite care ».  **Type of respite studed :**  « day respite centres » |
| Ryan, T., Noble, R., Thorpe, P., Nolan, M. (2008) Out and about: a valued community respite service. *Journal of Dementia Care*, 16(2), 34-35. | Expert opinion | To describe a respite service where people with dementia are accompanied to activities they enjoy in the community. |  | Level of evidence = 6 | UK  (Sheffield) | 2008 | The Community Dementia Support has been providing short breaks for carers of people with dementia since November 2000 | **Subject discussed :**  « The Community Dementia Support Service : aim to :  Provide short flexible respite provision in the community ; ensure that the focus for people with dementia is on participation in social life ; operate outside normal working hours (including weekends) ; ensure continuity between the family and support worker ; provide care by skilled and well supported staff. This nurse-led service comprises 14 support workers, one nurse practioner and one registred nurse, and provides 240 hours contact time per week to approximately 45 families »  **Type of respite studed :**  « institutionally-based services » |
| Shanley, C. (2006) Developing more flexible approaches to respite for people living with dementia and their carers. Journal of Alzheimer's Disease & Other Dementias, 21(4), 234-241. PMID: 16948287 | Expert opinion | This study of demetia respite services explores the notion of flexibility and then presents a comprehensive checklist that respite service providers can use to assess the flexibility of their service. |  | Level of evidence = 6 | USA | 2006 | No following period | **Subject discussed :**  « A checklist was made : a pratical tool for respite services to use in assessing how flexible they are in the way in which they provide respite. The service can use it as an overall assesment of the service by just ticking the boxes and getting an overall picture of how flexible the service seems. Alternaltively, they can invest more time and write in why they answered each statement the way they did. This will allow a more thorough analysis of how the service is working. A third use of the checklist could be to focus on parts of the checklist only if specific issues had already been identified as problems »  **Type of respite studed :**  « host family day respite, host family overnight respite, overnight stays at day centers, social outings as a form of respite, holiday programs, respite provided with intensive carer education and support, day center with extended hours, day center run in conjunction with In-home respite, programs for specific target groups, in-home occasional or emergency respite, residential respite in a noninstitutional setting, day respite offered by a nursing home, mobile respite service, respite combined with providing something special for the carers ans respite through a leisure buddy system. |
| Sorrell, JM., Cangelosi, PR. (2009) Caregiver burden or caregiver gain? *Journal of Psychosocial Nursing Ment Health Service,* 47(9),19-22. doi: 10.3928/02793695-20090730-04. PMID:19772247 | Expert opinion | To discusse about barriers and proposes suggestions for overcoming them. |  | Level of evidence = 6 | USA | 2009 | No following period | **Subject discussed :**  « this article discuss the burden of caregivers, respite institutions and barriers to the use of respite day centers  **Type of respite studed :**  « in-home assistance, day care center, nursing home, assisted living facilities » |
| Strang, VR.,Haughey, M. (1999) Respite--a coping strategy for family caregivers…including commentary by Gerdner LA and Teel CS with author response. *Western Journal of Nursing* Research, 21(4), 450-471. PMID: 11512165 | Qualitative phenomenological study | Explore how 10 family caregivers of persons with dementia experienced respite. | 10 participants | MMAT = 4/4 | Canada | 1999 | 2 months | **Method :**  « In this study ti was important to seek out the meaning of respite frome the caregivers perspectives and to hear the descriptions about their realities. The caregivers and the researcher focused on how caregiving was being experienced, how respite was perceived within that experience, and what allowed the caregivers to consider respite for themselves. In this study, 10 familiy caregivers were individually intervied twice about 2 months apart with each interview lasting about 90 minutes »  **Type of respite studed :**  « self-help group, informal community connections and various home care agencies » |
| Strang, VR., (2000) Caregiver respite: coming back after being away. *Perspectives: The Journal of the Gerontological Nursing Association*, 24(4), 10-20. PMID: 12026573 | Qualitative phenomenological study | To examinate how the caregiver's respite experience influenced their return to the responsabilities of carinf for their family members. | 20 participants | MMAT = 4/4 | Canada | 2000 | 2 months | **Method :**  « In this study, the caregivers and researcher together focused on the meaning of the respite experience as it influenced the return to the responsabilities of caring for them and how they determined the effectiveness of a respite experience for themselves. Twenty family caregivers were included in the study and were interviewed by a research assistant, a skilled qualitative research interviewer. Five caregivers were interviewed a second time about 60 to 90 minutes. All the interviews were transcribed verbatim »  **Type of respite studed :**  « home care agencies and inforrmal community connections » |
| Strang, VR., Haughey, M. (1998) Factors influencing the caregiver's ability to experience respite. *Journal of Family Nursing*, 4(3), 231-254. | Qualitative grounded theory | Describe the respite experience as a cognitive process of getting out of the caregiver world and into their own world. | 10 participants | MMAT = 4/4 | Canada | 1998 | 2 months | **Method :**  « the researcher and the caregivers focused on how the latter experienced caregiving, how they perceived respite within the experience, and what allowed them to accept the experience for themselves. In this study, 10 family caregivers were individually interviewed twice, about 2 months apart, with each lasting about 90 minutes. »  **Type of respite studed :**  « in-home personal care, day/night sitters, institutional respite beds and adult day programs. » |
| Stirling, C., Andrews, S., Croft, T., Vickers, J., Turner, P., Robinson, A. (2010) Measuring dementia carers' unmet need for services--an exploratory mixed method study. *BMC Health Services Research*, 10,122. doi: 10.1186/1472-6963-10-122. PMID:  20465782 PMCID:  PMC2875230 | Mixted study | To explore the link between measures of carer burden, service use, and carer's stated need. | 20 community-dwelling pairs of dementia carers and people with dementia | MMAT= 3/4 | Australia | 2010 | 12 weeks | **Method :**  « Four visits took place with each carer over 12 weeks, at weeks 1, 4, 8 and 12. During the first visit, as data collection  was occurring with carers, care recipients were seen by a psychologist in a separate location in their homes. Carers completed self-report measures on carer burden and stress (normative need measures), indicated their service wants (felt need measures), kept a service usage diary over the 12 week study period (expressed need measures), and participated in three semi-structured interviews, conducted at monthly intervals. Data was compared across participants. »  **Type of respite studed :**  « Practical assistance, in-home respite, out-of-home respite » |
| Townsend, D., Kosloski, K. (2002) Factors related to client satisfaction with community-based respite services. *Home Health Care Services Quarterly*, 21(3/4), 89-106. PMID: 12665073 | Quantitative descriptive | To identified factors related to client satisfaction with respite servcies. | 1183 participants | MMAT = 4/4 | USA  (South Carolina, Washington DC, Maine, Puerto Rico, Michigan, Ohio, Florida, Maryland,  Montana, California, Washington, North Carolina,  and Hawaii | 2002 | interviews were conducted a minimum  of six months after the initial use of respite | **Method :**  « phone interviews with family or friends who served as the primary  Caregiver. In some cases, language and cultural barriers required in-person interviews  by interviewers »  **Type of respite studed :**  « adult day care,  in-home, or institutional respite care » |
| Takai, Y., Yamamoto-Mitani, N., Okamoto, Y., Fukahori, H., Ko, A., Tanaka, M. (2013) Family caregiver strategies to encourage older relatives with dementia to use social services. *Journal of Advanced Nursing,* 69(12), 2675-2685. PMID: 23600994 | Qualitative grounded theory | To elicit the strategies used by Japanese family caregivers in the community to encourage older relatives with dementia to use adult daycare and respite stays. | 16 participants | MMAT = 4/4 | Japan | 2013 | Between 2005 - 2007 | **Method :**  « Data were collected through interviews. Participants spoke  freely about their experiences with care-giving, adult daycare and short respite. To gain further detail, we used  probing questions when necessary. Participants were asked additional questions in later interviews because patterns identified during comparative analysis of the earlier data produced new questions, which could be used to gain deeper insight into participant experiences. Interviews lasted between 1–2 hours and were recorded and transcribed verbatim. »  **Type of respite studed :**  « adult day care and respite stays » |
| Van Werkhooven, M. (1991) Respite care in the long-term care continuum. *Journal of Long-Term Care Administration*, 36-39. PMID: 10119226 | Expert opinion | A discussion about different respite care |  | Level of evidence = 6 | USA | 1991 | No following period | **Subject discussed :**  « this article talks about the different models of repit service and their benefits ».  **Type of respite studed :**  « adult day care, home aides and overnight stays, hospitals » |
| Vecchio, N., Fitzgerald, JA., Radford, K.,Fisher, R. (2016) The association between cognitive impairment and community service use patterns in older people living in Australia. *Health and Social Care in the Community*,24(3),321-33. doi: 10.1111/hsc.12212 PMID:  25754586 | Quantitative non-randomized (cross-sectionnal analytic study) | To better understand the relationship between cognitive impairment and the receipt of community care services. | 59352 participants | MMAT= 3/4 | Australia  (Queensland) | 2016 | 12 month period in 2007-2008 | **Method :**  «The analysis was based on the Australian HACC. The data consisted  of de-identified client information acquired from approximately 714 active service providers that included community, commercial, religious and charitable  organisations and state and local government  bodies. All service  providers that received HACC funding were required  to collect data on a quarterly basis and submit their  data to the National Data Repository for validation »  **Type of respite studed :**  « nursing care, allied healthcare, respite  care, home maintenance, counselling, assessment,  case management, day centre care, care co-ordination,  social support, domestic assistance and personal care » |

1. Quality Score MMAT or Level of Evidence (Tavares de Souza, M. et al. 2010). [↑](#footnote-ref-1)
